# Community differentiation of rhizosphere microorganisms and their responses to environmental factors at different development stages of medicinal plant *Glehnia littoralis*

**DOI:** 10.7717/peerj.14988

**Published:** 2023-03-06

**Authors:** Shuliang Liu, Jianxin Gao, Shimeng Wang, Weiwei Li, Ailan Wang

**Affiliations:** Ludong University, Yantai City, Shandong Province, China

**Keywords:** Bacterial and fungal communities, High-throughput sequencing, 16S, ITS, Diversity of rhizosphere microbes

## Abstract

Rhizosphere microorganisms play a key role in affecting plant quality and productivity through its interaction with plant root system. To figure out the bottleneck of the decline of yield and quality in the traditional Chinese medicinal herbs *Glehnia littoralis* they now encounter, it is important to study the dynamics of rhizosphere microbiota during the cultivation of *G. littoralis*. In the present study, the composition, diversity and function of rhizosphere microbes at different development stages of *G. littoralis*, as well as the correlation between rhizosphere microbes and environmental factors were systematically studied by high-throughput sequencing. There were significant differences between the rhizosphere microbes at early and middle-late development stages. More beneficial bacteria, such as Proteobacteria, and more symbiotic and saprophytic fungi were observed at the middle-late development stage of *G. littoralis*, while beneficial bacteria such as Actinobacteria and polytrophic transitional fungi were abundant at all development stages. The results of redundancy analysis show that eight environmental factors drive the changes of microflora at different development stages. pH, soil organic matter (SOM) and available phosphorus (AP) had important positive effects on the bacterial and fungal communities at the early development stage; saccharase (SC) and nitrate nitrogen (NN) showed significant positive effects on the bacterial and fungal communities at the middle and late stages; while urease (UE), available potassium (AK), and alkaline phosphatase (AKP) have different effects on bacterial and fungal communities at different development stages. Random forest analysis identified 47 bacterial markers and 22 fungal markers that could be used to distinguish *G. littoralis* at different development stages. Network analysis showed that the rhizosphere microbes formed a complex mutualistic symbiosis network, which is beneficial to the growth and development of *G. littoralis*. These results suggest that host development stage and environmental factors have profound influence on the composition, diversity, community structure and function of plant rhizosphere microorganisms. This study provides a reference for optimizing the cultivation of *G. littoralis*.

## Introduction

The plant root system, rhizosphere microorganisms and rhizosphere soil constitute a plant rhizosphere microecosystem. In this microecosystem, biotic factors (plant genotypes, plant developmental stages, invasive pathogenic microorganisms) and abiotic factors (soil composition, soil management and climatic conditions) in soil influence the composition, diversity, structure and function of rhizosphere microbial communities (Vacheron et al., 2013; Gouda et al., 2018; Li et al., 2020; Ma et al., 2021). In turn, rhizosphere microorganisms influence plant root exudates and even plant metabolome (Badri et al., 2013), which involves improving plant physiology and resistance to pathogens through various mechanisms (Zakry et al., 2012; Bai et al., 2022), affecting plant health and growth (Trivedi et al., 2020; Xiong et al., 2021). Rhizosphere microorganisms also affect soil evolution and play a vital role in the conversion of poor soil and low-quality soil into cultivable soil (Gouda et al., 2018). In summary, multitrophic interactions between plants, microorganisms and environmental factors lead to the formation of complex symbiotic networks in rhizosphere microecosystem. This symbiotic network dynamically affects rhizosphere communities and alters plant phenotypes (Lu et al., 2018).

Beneficial rhizosphere microorganisms can enhance plant roots’ vitality, promote plant growth, increase plant yield and improve resistance to phytopathogens (Razavi et al., 2016). Conversely, harmful microorganisms can lead to plant disease, the inhibition of root growth, the suppression of plant growth, and crop failure (Kremer, 2006). The study of the symbiosis and interactions between medicinal plants, environmental factors and microbes can help regulate the microbiota of medicinal plants themselves and their surroundings, contribute to the goal of high quality and high yield of medicinal plants (Karthikeyan et al., 2008; Khamna, Yokota & Lumyong, 2009; Köberl et al., 2013; Solaiman & Anawar, 2015; Kushwaha et al., 2020). Therefore, it is of great significance to study the dynamics of rhizosphere microbiota in the cultivation of medicinal herbs.

The traditional Chinese medicinal herb “Beishashen” is the swelling root of perennial herb *Glehnia littoralis* Fr. Schmidt ex Miq (Shan & She, 1992). It contains a variety of coumarin compounds and alkaloids, and has a variety of activities such as antibacterial and anti-inflammatory (Taesook et al., 2010; Jing et al., 2021), so it is widely used in China and Southeast Asia. Due to the endangered status of the wild *G. littoralis* resources (Fu, 1992), the medicinal herb “Beishashen” on the market mainly comes from human cultivation. In China, *G. littoralis* has been cultivated for more than 600 years. Due to its special habitat requirements, it can only grow in sandy soil, which makes its planting area relatively fixed. Long-term planting in fixed areas results in typical negative plant-soil feedback (NPSF), leading to a decline in the yield and quality of the medicinal herbs (Cesarano et al., 2017; Yuan et al., 2022). Interactions between rhizosphere microbiota and plants affect plant health and productivity, and this process is very important in medicinal plant cultivation (Trivedi et al., 2020; Xiong et al., 2021). Given the importance of rhizosphere microbes, the study of rhizosphere microbiota may provide a way to improve the yield and quality of the medicinal herbs. Relevant microbial research has attracted the attention of the researchers, but so far, only the bacteriostatic activity of endophytic fungi and related studies have been reported (Huo et al., 2020).

To provide a basis for the interpretation and utilization of beneficial rhizosphere microbial resources, rhizosphere microbes of *G. littoralis* in genuine producing areas were analyzed by high-throughput sequencing technique. The composition, diversity, function, and dynamics of rhizosphere microorganisms at different development stages of *G. littoralis*, as well as the correlation between rhizosphere microorganisms and environmental factors, were investigated, hoping to provide reference data for optimizing cultivation of Chinese medicinal herb “Beishashen”.

## Materials and Methods

### Sampling

All the samples were collected from Haiyang City (37°01′27.04″N, 120°44′53.71″E), Shandong Province, China, one of the main cultivation areas of *G. littoralis*. As a perennial plant, *G. littoralis* is usually harvested within a year when used as a Chinese medicinal herb. Therefore, samples growing for only one year in three growing seasons (Spring, Summer and Autumn) were collected according to the phenological stages, representing the seedling stage, the vigorous growth stage and the harvesting stage, respectively. In a 2 m × 2 m quadrat, three samples were collected on the diagonal of the quadrat as triplicates of each development stage. Since the root system at the seedling stage is small, in order to collect enough rhizosphere soil, the rhizosphere soil of 2–3 *G. littoralis* seedlings on the diagonal of each quadrat was collected as a replicate. The collected rhizosphere soil samples were quickly transported to the laboratory at low temperatures. Some soil samples were kept in the shade to test soil physiochemical properties, and the rest was stored at −80 °C for high-throughput sequencing. Field experiments were approved by Ludong University (project number 20210301).

### Measurement of soil physiochemical properties

Soil samples with roots and debris removed using a 2 mm sieve were air-dried and stored at 4 °C for use. Fourteen common environmental factors in soil were detected according to previous studies, including available phosphorus (AP), available potassium (AK), ammonium nitrogen (AN), soil organic matter (SOM), total organic carbon (TOC) (Bao, 2000), nitrate nitrogen (NN) (Sun et al., 2016), Saccharase (SC), Urease (UE), alkaline phosphatase (AKP) (Guan, 1986); total nitrogen (TN), total hydrogen (TH), total carbon (TC), and total sulfur (TS) were detected by Elemental Analyzer (Elementar vario EL cube Elemental Analyzer; Elementar, Langenselbold, Germany) and the pH of soil samples was determined in 1:2.5 soil-water suspension using pH meter (Sartorius PB-10). The saccharase activity was expressed as mg glucose·d^−1^·g^−1^ soil, the urease activity was expressed as mg NH_3_-N·d^−1^·g^−1^ soil and the alkaline phosphatase activity was expressed as mg P_2_O_5_·2h^−1^·g^−1^ soil.

### DNA extraction and high-throughput sequencing

The total DNA of rhizosphere microorganisms was extracted using HiPure Soil DNA Kits (Tiangen, Beijing, China) according to the manufacturer’s protocols. The extracted DNA was used for PCR amplification. For bacteria, the 16S V3–V4 region of the ribosomal RNA gene was amplified using primers 338F (5′-ACTCCTACGGGAGGCAGCA-3′) (Huse et al., 2008), and 806R (5′- GGACTACHVGGGTATCTAAT-3′) (Caporaso et al., 2011). The PCR program is as following: 95 °C for 5 min, followed by 25 cycles at 95 °C for 30 s, 50 °C for 30 s, and 72 °C for 40 s and a final extension at 72 °C for 7 min. For fungi, the ITS (internal transcribed spacer) region of the ribosomal RNA gene was amplified using primers ITS-F (5′-CTTGGTCATTTAGAGGAAGTAA-3′) (Gardes & Bruns, 1993) and ITS-R (5′-GCTGCGTTCTTCATCGATGC- 3′) (White et al., 1990) by PCR (95 °C for 5 min, followed by 25 cycles at 95 °C for 1 min, 50 °C for 30 s, and 72 °C for 1 min and a final extension at 72 °C for 7 min). PCR products were confirmed by 1.8% agarose gel electrophoresis and purified using VAHTSTM DNA Clean Beads (Vazyme, Nanjing, China). A library was constructed, the Solexa PCR product was purified, and the sequenced library was constructed after quantification and homogenization. The DNA was purified with the Monarch DNA Gel Extraction Kit (Hongyue, Beijing, China) and then subjected to high-throughput sequencing on Illumina Novaseq 6000 (Biomarker Technologies, Beijing, China). The raw sequencing data has been uploaded to the NCBI Sequence Read Archive (SRA) database (BioProject: PRJNA903756). Three biological replicates of each stage were sequenced.

### Data analysis

Raw data were first filtered by Trimomatic (v0.33) (Bolger, Lohse & Usadel, 2014). Primer sequences were then identified and removed by Cutadapt 1.9.1 (Martin, 2011), resulting in high-quality reads without primer sequences. Based on overlapping sequences, high-quality reads were assembled by FLASH (v1.2.7) (Magoc & Salzberg, 2011), which generated clean reads. Chimeric sequences were identified and removed by UCHIME (v4.2) (Edgar et al., 2011), generating effective reads. The effective reads were then clustered with Usearch software (v10) at a similarity level of 97.0% to obtain operational taxonomic units (OTUs) tables (Edgar, 2013). QIIME2 software (V2020.6) was used to evaluate the Alpha diversity and Beta diversity of samples (Bolyen et al., 2019). Taxonomic annotation was carried out based on SILVA database (release 138) (Quast et al., 2013), and the community composition of each sample was counted at six levels (phylum, class, order, family, genus, species). PICRUSt2 (Douglas et al., 2019) was used to perform bacterial function prediction analysis, and FUNGuild (Nguyen et al., 2016) was used to predict the nutritional and functional groups of the fungal communities. Biomarkers were extracted using the RandomForest (Breiman, Last & Rice, 2006) package in R (V4.2) (Ihaka & Gentleman, 1996). Network analysis and redundancy analysis (RDA) were performed to assess the interaction between rhizosphere microorganisms and environmental factors. Gephi software (V0.9.7) (Bastian, Heymann & Jacomy, 2009) was used for network analysis based on Pearson’s correlation coefficient. RStudio (V2022.07.1) was used for the RDA analysis and the heatmap (Racine, 2012). The STAMP (V2.1.3) software (Parks et al., 2014) was used to analyze the functional differences between rhizosphere microbiota. Box maps, abundance maps and PcoA analysis were performed using the online mapping tool imageGP (http://www.ehbio.com/Cloud_Platform/front/#/) (Chen, Liu & Huang, 2022). Venn diagrams was produced using the online mapping tool jvenn (http://www.bioinformatics.com.cn/static/others/jvenn/example.html) (Bardou et al., 2014).

## Results

### Microbial community composition, diversity and structure

#### Feature of operational taxonomic units (OTUs)

**Figure 1:**
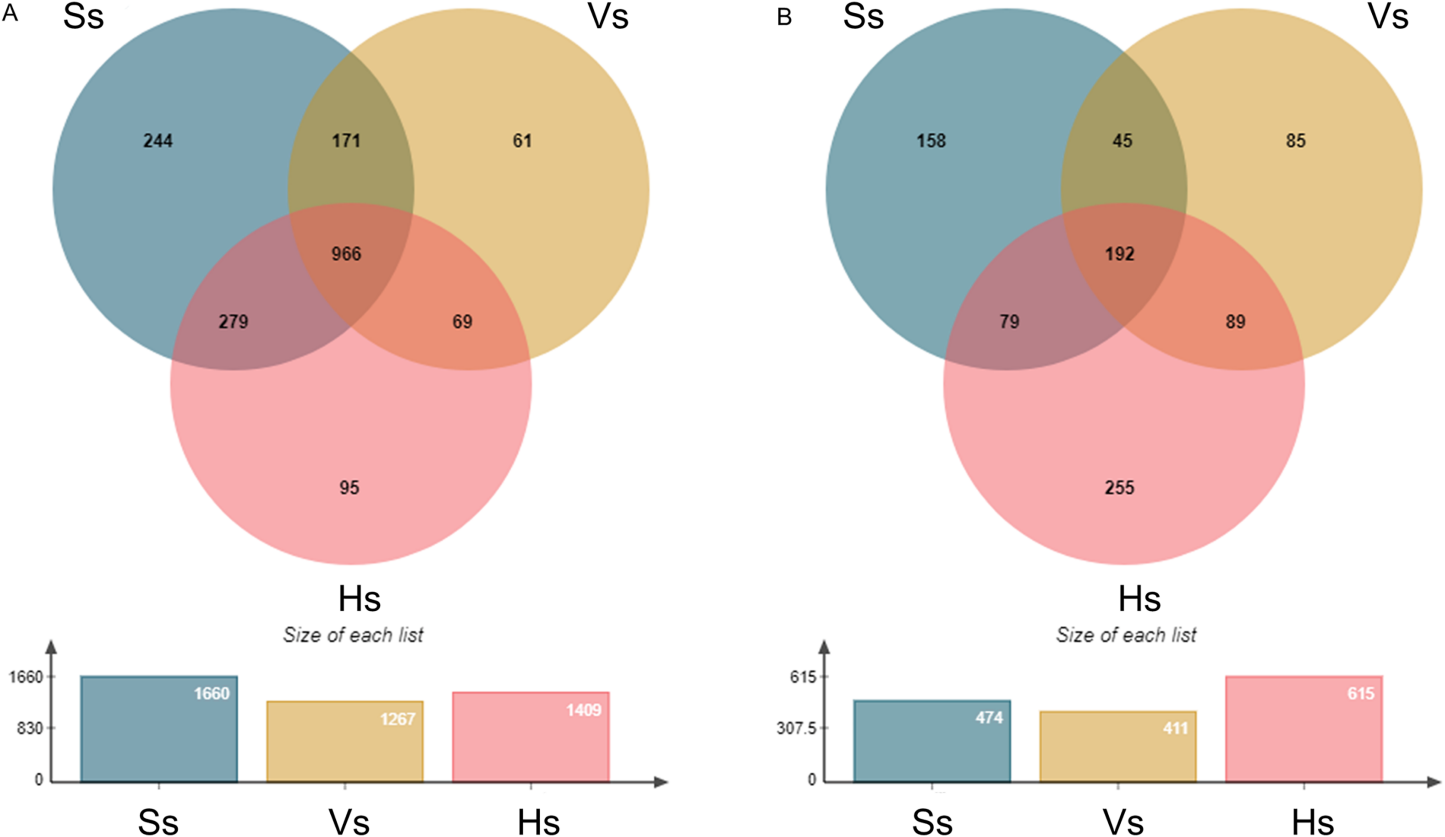
Venn diagram of OTU distribution at different development stages of *Glehnia littoralis*. (A) Bacteria community; (B) fungi community; Ss, the seedling stage; Vs, the vigorous growth stage; Hs, the harvesting stage.

The operational taxonomic units (OTUs) of rhizosphere bacteria and fungi were obtained from all the samples at the three growth stages (Fig. 1). A total of 1,885 bacterial OTUs were identified in rhizosphere soil of three growth stages, including 1,660 OTUs at the seedling stage, 1,267 OTUs at the vigorous stage, and 1,409 OTUs at the harvesting stage, accounting for 88.06%, 67.21% and 74.75% of the total bacterial OTUs at each growth stage, respectively. A total of 966 OTUs were shared by three different growth stages of *G. littoralis*, 244 OTUs specific to the seedling stage, 61 OTUs specific to the vigorous growth stage, and 95 OTUs specific to the harvesting stage (Fig. 1 A). A total of 903 fungal OTUs were found in the soil at all growth stages, including 474 OTUs at the seedling stage (52.49%), 411 OTUs at the vigorous growth stage (45.51%), 615 OTUs at the harvesting stage (68.11%). A total of 192 OTUs were found to be shared by three different growth stages of *G. littoralis*, 158 OTUs specific to the seedling stage, 85 OTUs specific to the vigorous growth stage, and 255 OTUs specific to the harvesting stage (Fig.1B). The largest number of OTUs of bacteria was at the seedling stage, and the largest number of OTUs of fungi was at the harvesting stage (Figs. 1A and 1B). Similarly, the specific OTUs at the two stages were also in the largest number (Figs. 1A and 1B).

#### Variation in the abundance and diversity of rhizosphere bacteria and fungi

**Figure 2:**
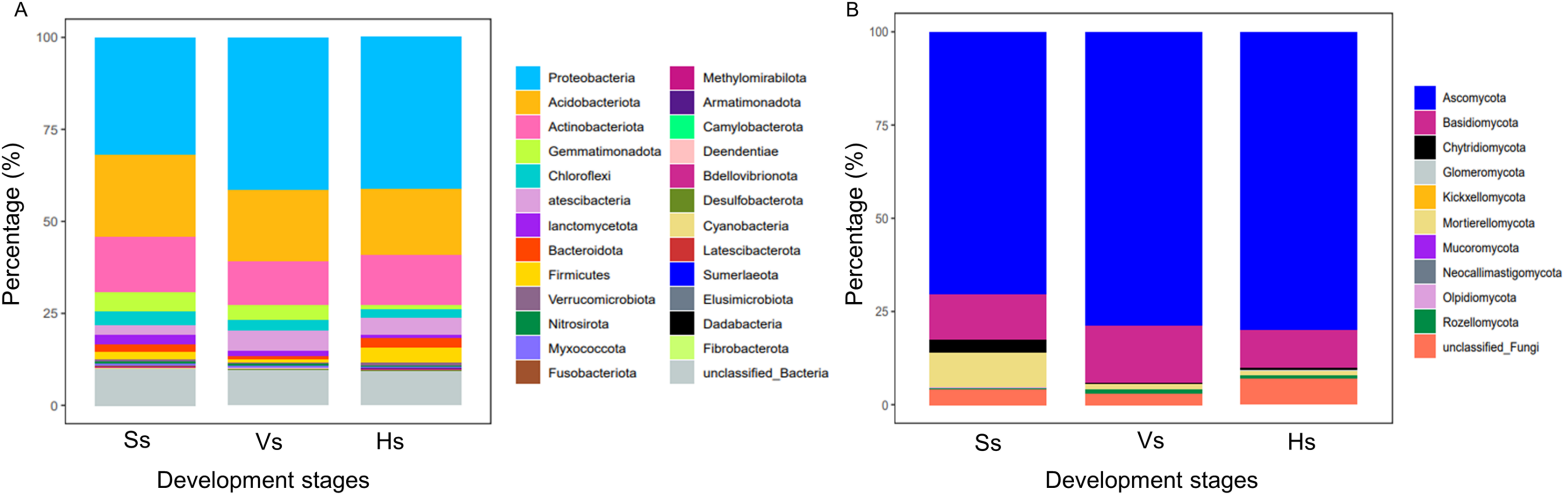
Relative species abundance of bacterial and fungal communities at phylum level. (A) Bacteria community; (B) fungi community; Ss, the seedling stage; Vs, the vigorous growth stage; Hs, the harvesting stage.

The bacterial OTUs were clustered into 25 phyla and 479 genera. At phylum level, the relative abundance of bacteria showed no obvious difference between the bacterial communities at the vigorous growth stage and harvesting stage, but obvious differences were found between the bacteria at the two stages and those at the seedling stage (Fig. 2A). Proteobacteria was the major component of each bacterial community, representing 31.94%, 41.36% and 41.15% of the total species in each community at each development stage, respectively; Acidobacteriota was the second most abundant phylum after Proteobacteria, which happened in 22.30%, 19.61% and 17.94% of all the samples at each development stage, respectively; except for Proteobacteria and Acidobacteriota, Actinobacteria was the main phylum with abundance above 10%, which happened in 15.02%, 11.84% and 13.66% of the samples at each development stage, respectively (Table S1). The fungal OTUs were assigned to 10 phyla and 312 genera. Similar to bacteria community, the abundance of dominant phylum in fungi community showed no obvious difference between the vigorous growth stage and the harvesting stage, but there were obvious differences between the fungi at the two stages and those at the seedling stage (Fig. 2A). Ascomycota was the most enriched phylum representing 70.64%, 78.98%, and 80.14% of the total species at each growth stage and Basidiomycota was the second abundant phylum after Ascomycota, which happened in 11.93%, 15.2% and 9.88% of the samples at each development stage (Table S2).

#### Diversity of rhizosphere microbiota

**Figure 3:**
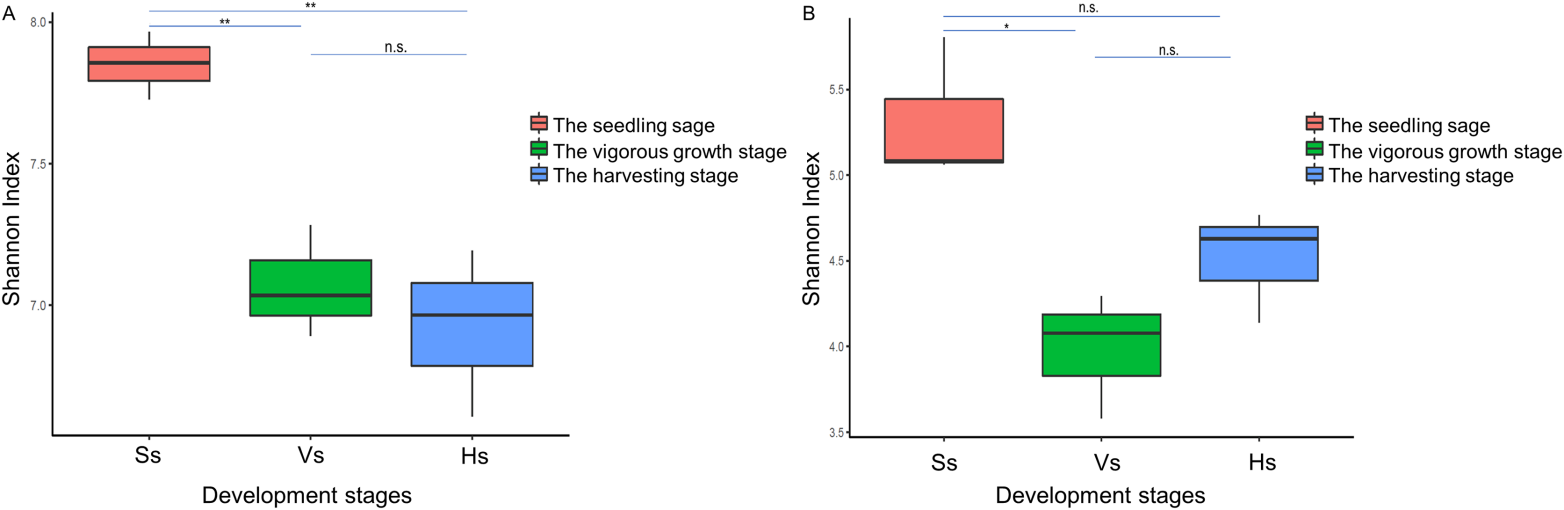
Shannon index of rhizosphere microbial community at different development stages of *G. littoralis*. (A) Bacteria community; (B) fungi community; Ss, the seedling stage; Vs, the vigorous growth stage; Hs, the harvesting stage. *P < 0.05; **P < 0.01; n.s., no significant difference.

The alpha diversity of the bacterial community of *G. littoralis* showed significant differences between the seedling stage and the vigorous growth and harvesting stages, while there was no significant difference between the vigorous growth stage and the harvesting stage (Table 1, Fig. 3). The bacterial community’s richness (ACE and Chao1) and diversity (Shannon index) were the highest at the seedling stage, and followed by the vigorous growth stage and the harvesting stage (Table 1). There was no significant difference in ACE, Chao1 and Shannon index between rhizosphere bacterial communities at the vigorous growth stage and the harvesting stage (Table 1). In addition, the richness and diversity of bacterial communities showed a gradually decreasing trend with the different development stages of *G. littoralis* (Table 1). The alpha diversity analysis results of fungi community are also listed in Table 1; it is shown that the fungi community’ richness (ACE and Chao1) at the harvesting stage was the highest, followed by the seedling stage and the vigorous growth stage (Table 1). This result was different from that of the bacterial community. The Shannon index of fungi community at the seedling stage was the highest and significantly higher than that at the vigorous growth stage. The Shannon index of fungi community at the harvesting stage was slightly higher than that at the vigorous growth stage, and the difference was small and not significant.

**Table 1:** The α-diversity of rhizosphere microbes at different development stages.

Sample ID		ACE	Chao1	Shannon	Coverage
Bacteria	Ss	1,562.05a	1,566.4a	7.8498a	0.9945
	Vs	1,207.83b	1,127.97b	7.0705b	0.9969
	Hs	1,172.68b	1,158.76b	6.9217b	0.9967
Fungi	Ss	365.98ab	343.26ab	5.3165a	0.9996
	Vs	313.45b	309.71b	3.9844b	0.9994
	Hs	524.99a	447.14a	4.5112ab	0.9994

**Note:**

Ss, the seedling stage; Vs, the vigorous growth stage; Hs, the harvesting stage. Different lowercase letters indicate significant differences (*P* < 0.05) between groups.

**Figure 4:**
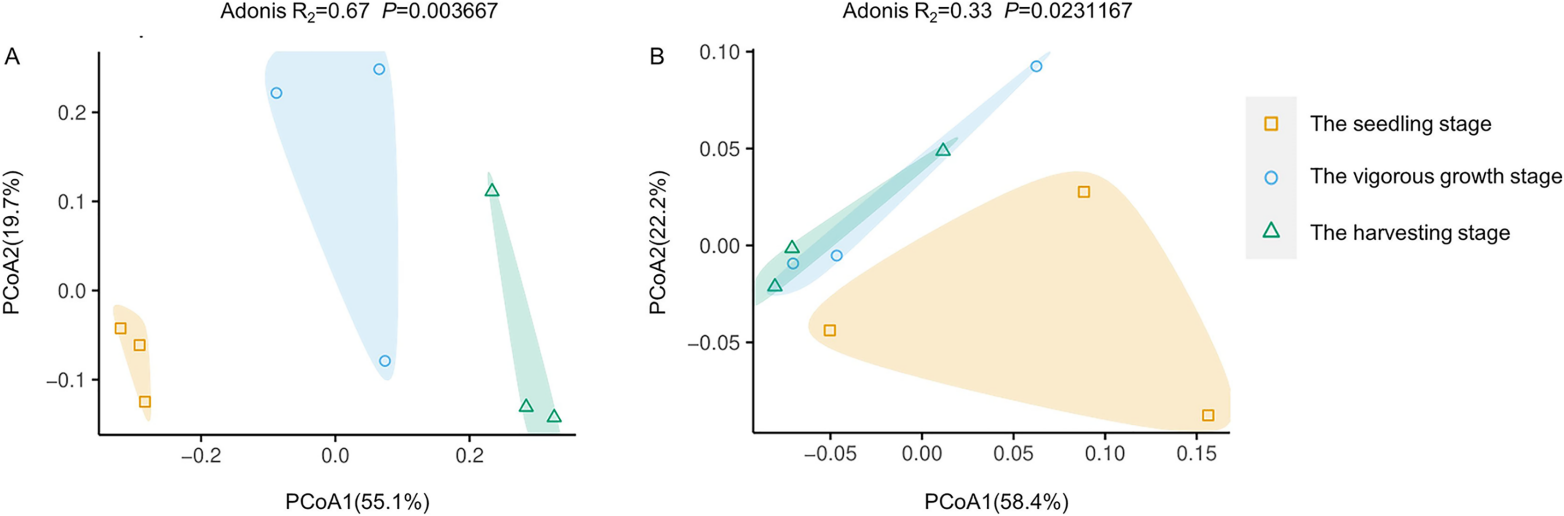
Principal coordinate analysis (PcoA) of rhizosphere microbial communities at different development stages of *G. littoralis*. (A) Bacteria community; (B) fungi community.

PCoA analysis based on Bray-Curtis distances was performed to demonstrate changes in rhizosphere microbial community structure at different development stages. Both rhizosphere bacteria and rhizosphere fungi were divided into two groups (Fig. 4). One group included only microbiota at the seedling stages, and the other group included microbiota at the vigorous growth stage and the harvesting stage. This was consistent with the results of alpha diversity analysis, indicating that the rhizosphere microorganisms at the seedling stage are different from rhizosphere microorganisms at other development stages.

### Characteristics of rhizosphere microbial function at different development stages

**Figure 5:**
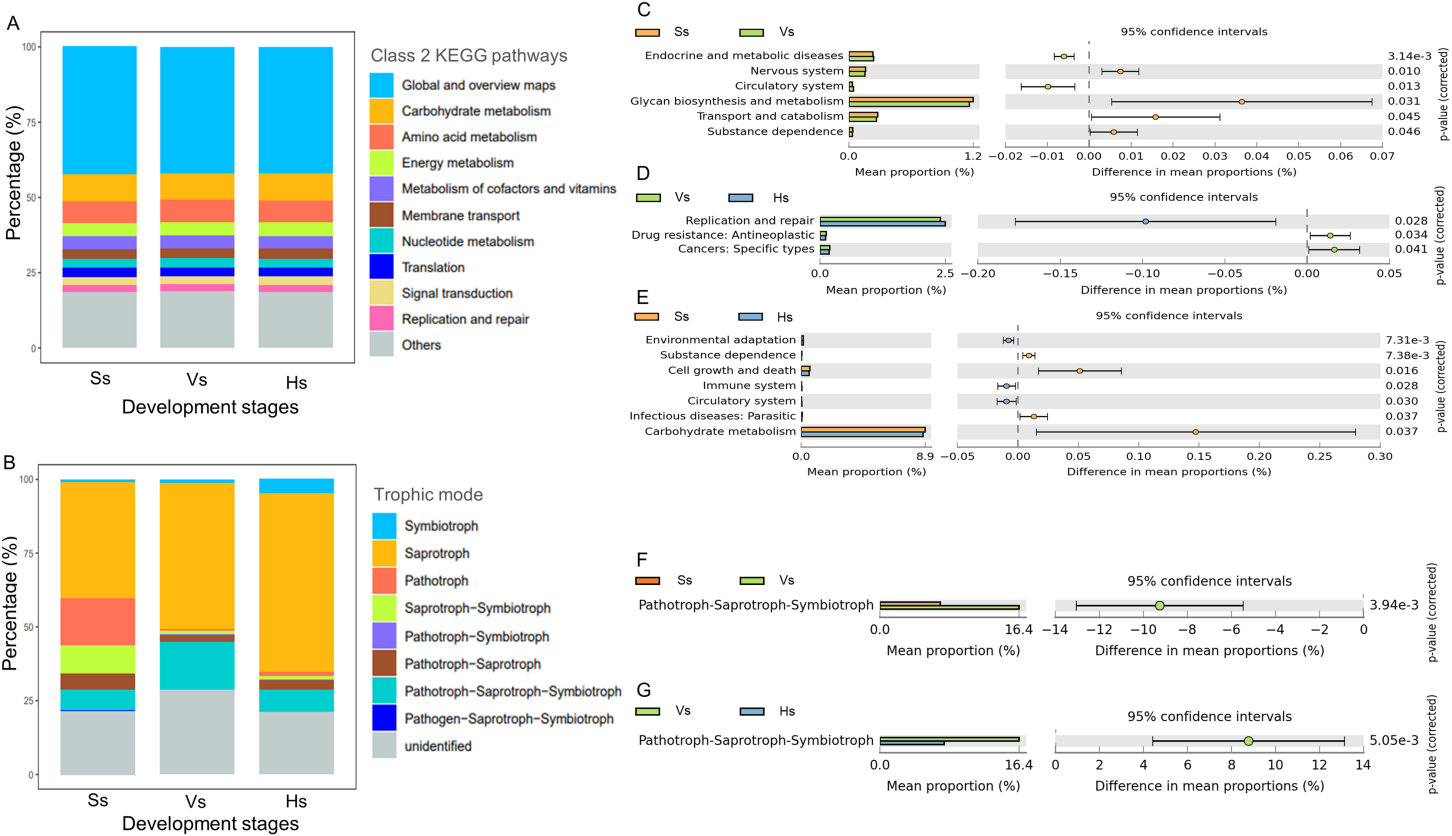
The main functional diversity and functional differences of rhizosphere microbial communities at different development stages of *G. littoralis*. (A) Bacteria community; (B) fungi community; (C–E) functional difference analysis of bacterial community; (F and G) functional difference analysis of fungal community.

There was no significant difference in the abundance of the major function at class 2 KEGG pathways in the microbiota of three development stages (Fig. 5A). The dominant 10 functions of bacteria include “Global and overview maps”, “Carbohydrate metabolism”, “Amino acid metabolism”, “Energy metabolism”, “Metabolism of cofactors and vitamins”, “Membrane transport”, “Nucleotide metabolism”, “Translation”, “Signal transduction” and “Replication and repair” (Fig. 5A). “Global and overview maps” was the main component, accounting for 42.13–42.32% (Table S3).

Comparison analysis of the functions between the rhizosphere bacteria of *G. littoralis* at three development stages showed that six, three and seven levels of functions were significantly different between the bacteria at the seedling stage and the vigorous growth stage, the vigorous growth stage and the harvesting stage, and the seedling stage and the harvesting stage, respectively (Figs. 5C–5E). From the seedling stage to the vigorous growth stage, the function of “Circulatory system” and “Endocrine and metabolic diseases” increased significantly, while “Glycan biosynthesis and metabolism”, “Transport and catabolism”, “Nervous system” and “Substance dependence” showed a significant decrease (Fig. 5C). Comparison of functions between the vigorous growth stage and the harvesting stage (Fig. 5D) showed a marked increase in “Replication and repair”, while “Drug resistance” and “Cancers” presented a marked decline. From the vigorous growth stage to the harvesting stage (Fig. 5E), the functions of “Cell growth and death”, “Carbohydrate metabolism”, “Infectious diseases: parastitic” and “Substance dependence” significantly decreased, and the functions of “Circulatory system”, “Immune system” and “Environmental adaptation” significantly increased.

Eight trophic modes were found in the fungi at three development stages (Fig. 5B), including symbiotroph, saprotroph, pathotroph, saprotroph-symbiotroph, pathotroph-symbiotroph, pathotroph-saprotroph, pathotroph-saprotroph-symbiotroph, and pathogen-saprotroph-symbiotroph. Saprotroph was the main trophic mode, accounting for 39.30–60.59% of the total fungi at each stage, and its relative abundance gradually increased with the development stages of *G. littoralis* (Fig. 5B, Table S4). In addition, pathotroph-saprotroph-symbiotroph was the second trophic mode after Saprotroph, accounting for 7.11–16.36% of fungi at each stage (Table S4).

Functional differences analysis showed that only the abundance of pathotroph-saprotroph-symbiotroph were significantly different at different development stages, and revealed an increasing trend and then a decreasing trend with the development of *G. littoralis* (Figs. 5F and 5G).

Finally, the PCA analysis of the functions at the three development stages was performed, and distinct differentiation was observed between the functions at the seedling stage and those at the vigorous growth and harvesting stages (Fig. S1).

### Microbial biomarkers in the rhizosphere at different development stages

**Figure 6:**
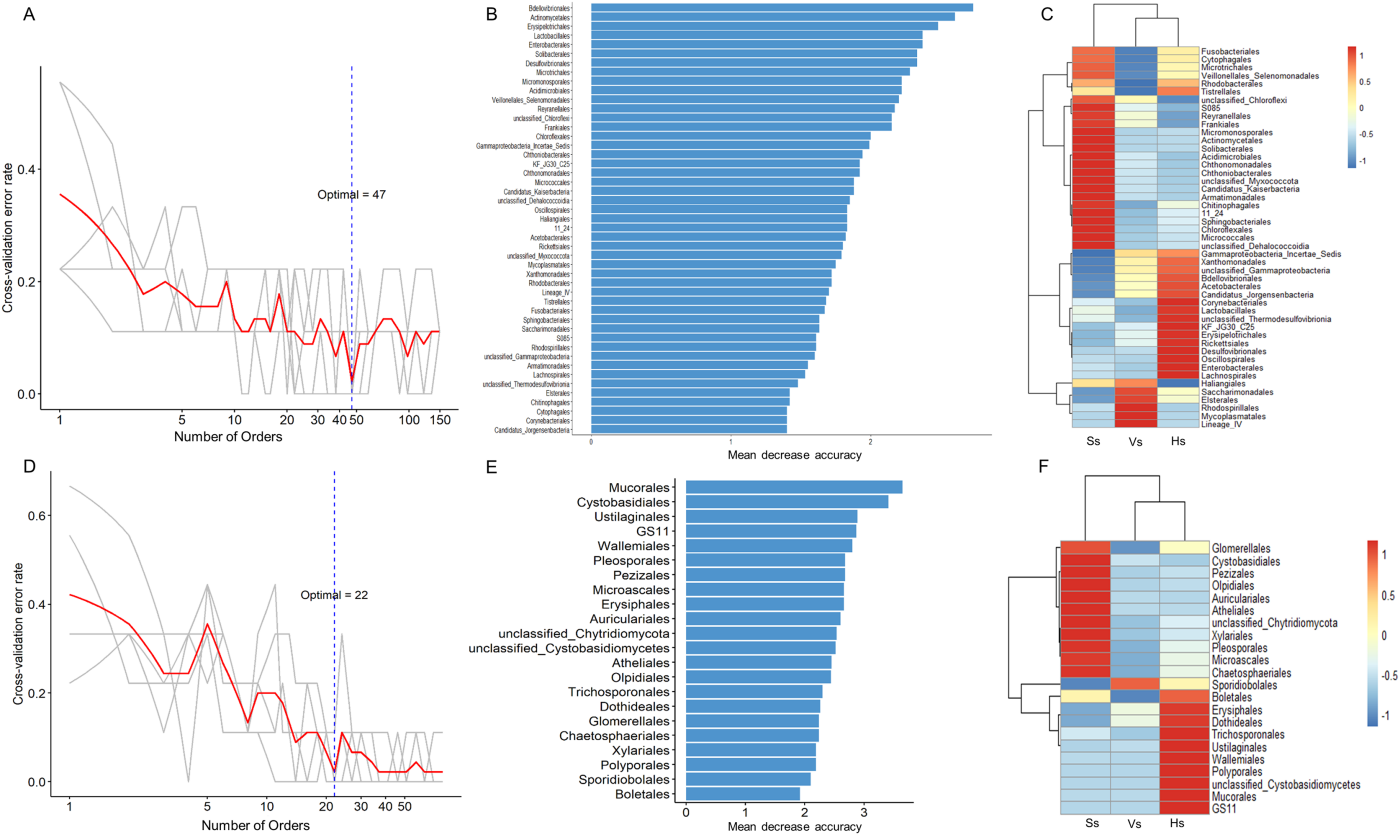
Bacterial and fungal biomarkers at order level at different development stages of *G. littoralis*. (A) The 10-fold cross-validation error and the identified numbers of bacterial biomarkers; (B) 47 bacterial biomarkers identified by Random Forest algorithm; the biomarkers are ranked in descending order according to importance to the accuracy of the model; (C) heat map showing the relative abundances of the 47 bacterial biomarkers against *G. littoralis* development time; (D) the 10-fold cross-validation error and the identified numbers of fungal biomarkers; (E) 22 fungal biomarkers identified by the random forest algorithm; the biomarkers are ranked in descending order according to importance to the accuracy of the model; (F) heat map showing the relative abundances of the 22 fungal biomarkers against *G. littoralis* development time. Ss, the seedling stage; Vs, the vigorous growth stage; Hs, the harvesting stage.

The random forest method was used to identify microbial biomarkers in the rhizosphere of *G. littoralis*. The optimal model of rhizosphere bacteria and fungi (Figs. 6A and 6D) was established based on the cross-validation results. The biomarkers identified in the samples were sorted according to their importance to the community (Mean Decrease Accuracy, MDA) from large to small to obtain a histogram (Figs. 6B and 6E). A heatmap analysis of rhizosphere microbes at different development stages was also performed (Figs. 6C and 6F). At bacterial order level, 47 important biomarkers were identified (Figs. 6A and 6B). These biomarkers can be used to distinguish samples at different stages accurately (Fig. 6C). The five orders with the greatest impact on bacterial community were Bdellovibrionales, Actinomycetales, Erysipelotrichales, Enterobacterales, and Lactobacillales (Fig. 6B). At fungal order level, 22 important biomarkers were identified (Figs. 6D and 6E). These biomarkers were also able to accurately distinguish samples at different development stages. The top five orders were Mucorales, Cystobasidiales, Ustilaginales, GS11, Wallemiales (Fig. 6E). The clustering results showed that both the bacterial and fungal biomarkers at three development stages clustered into two branches (Figs. 6C and 6F), one was composed with the microbiota at the seedling stage, another was composed with the microbiota at the vigorous growth stage and at the harvesting stage, which was consistent with the results of the above PcoA analysis (Figs. 4A and 4B).

### The interactions of microbes-microbes and microbes-environmental factors at different development stages

**Figure 7:**
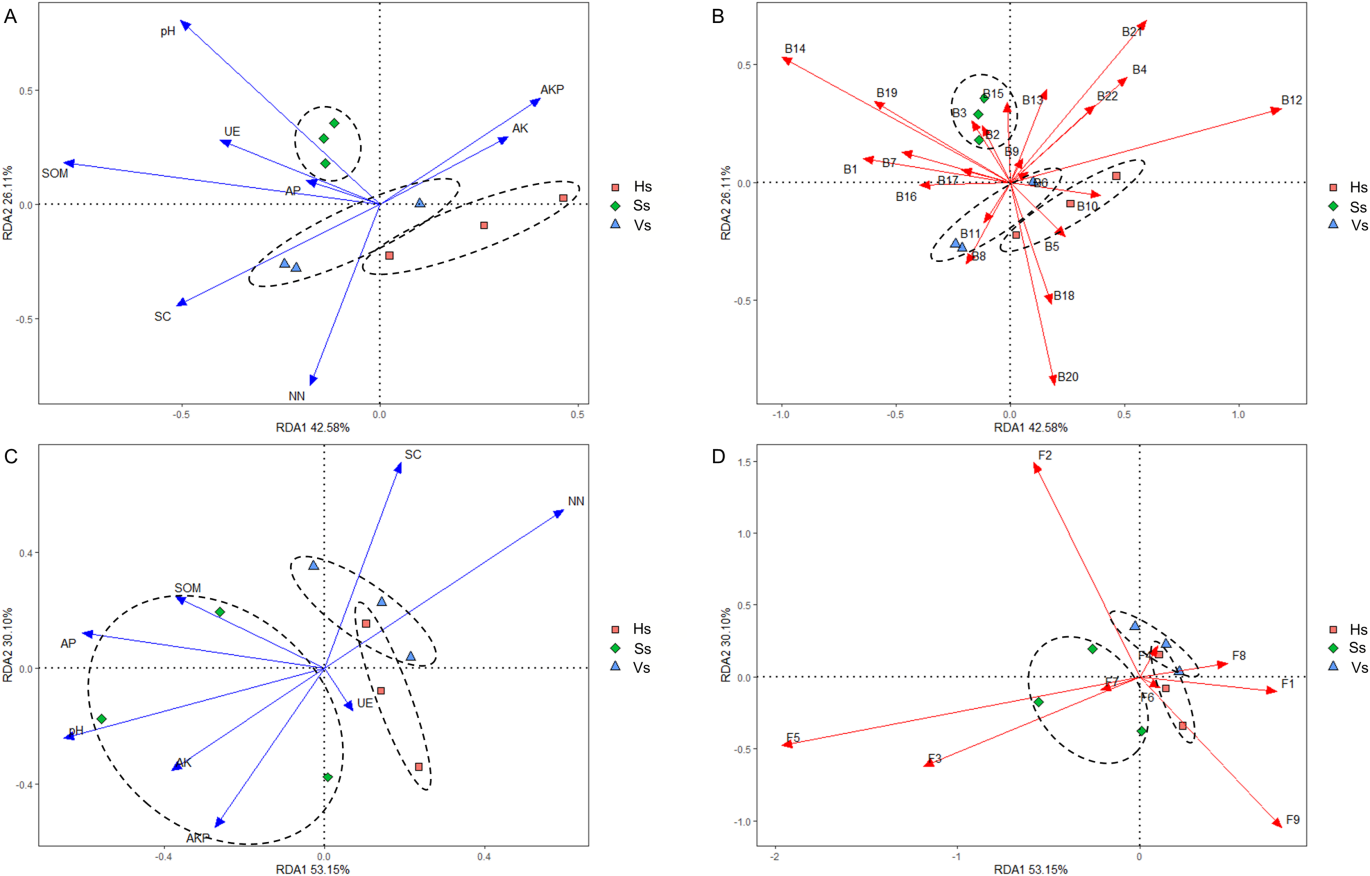
Redundancy analysis (RDA) of rhizosphere microbes-microbes-environmental factors. (A) Interactions of environmental factors with bacterial community at all development stages; (B) major bacteria at different development stages at the phylum level; (C) interactions of environmental factors with fungal community at all development stages; (D) major fungi at different development stages at the phylum level. Ss, the seedling stage; Vs, the vigorous growth stage; Hs, the harvesting stage. B1, Acidobacteriota; B2, Actinobacteriota; B3, Armatimonadota; B4, Bacteroidota; B5, Bdellovibrionota; B6, Campylobacterota; B7, Chloroflexi; B8, Cyanobacteria; B9, Dependentiae; B10, Desulfobacterota; B11, Elusimicrobiota; B12, Firmicutes; B13, Fusobacteriota; B14, Gemmatimonadota; B15, Methylomirabilota; B16, Myxococcota; B17, Nitrospirota; B18, Patescibacteria; B19, Planctomycetota; B20, Proteobacteria; B21, unclassified Bacteria; B22, Verrucomicrobiota; F1, Ascomycota; F2, Basidiomycota; F3, Chytridiomycota; F4, Glomeromycota; F5, Mortierellomycota; F6, Mucoromycota; F7, Olpidiomycota; F8, Rozellomycota; F9, unclassified_Fungi.

Redundancy analysis (RDA) revealed that eight environmental factors drove changes in the microbiome at different development stages (Figs. 7A and 7C). In the rhizosphere bacterial community, soil organic matter (SOM), pH, urease (UE), and available phosphorus (AP) showed a positive correlation with the bacterial community at the seedling stage; saccharase (SC) and nitrate nitrogen (NN) were positively correlated with the bacterial community at the vigorous growth stage and the harvesting stage; alkaline phosphatase (AKP) and available potassium (AK) impacted the bacterial community at all stages (Fig. 7A). The bacteria that played a major role at the seedling stage were Acidobacteriota, Actinobacteriota, Armatimonadota, Bacteroidota, Chloroflexi, Dependentiae, Firmicutes, Fusobacteriota, Gemmatimonadota, Methylomirabilota, Nitrospirota, Planctomycetota, and Verrucomicrobiota; The main bacteria at the vigorous growth and the harvesting stages were Proteobacteria, Patescibacteria, Bdellovibrionota, Campylobacterota, Cyanobacteria, Desulfobacterota, Elusimicrobiota, and Myxococcota (Fig. 7B). In the rhizosphere fungal community, SOM, AP, pH, AK, AKP and UE were the main influencing factors at the seedling stage, while SC and NN were the main influencing factors at the vigorous growth and harvesting stages (Fig. 7C). Chytridiomycota, Mortierellomycota, Olpidiomycota were the main compositions of fungal community at the seedling stage, and Ascomycota, Basidiomycota, Glomeromycota, Mucoromycota, and Rozellomycota were the main compositions of fungal community at the vigorous growth and harvesting stages (Fig. 7D).

**Figure 8:**
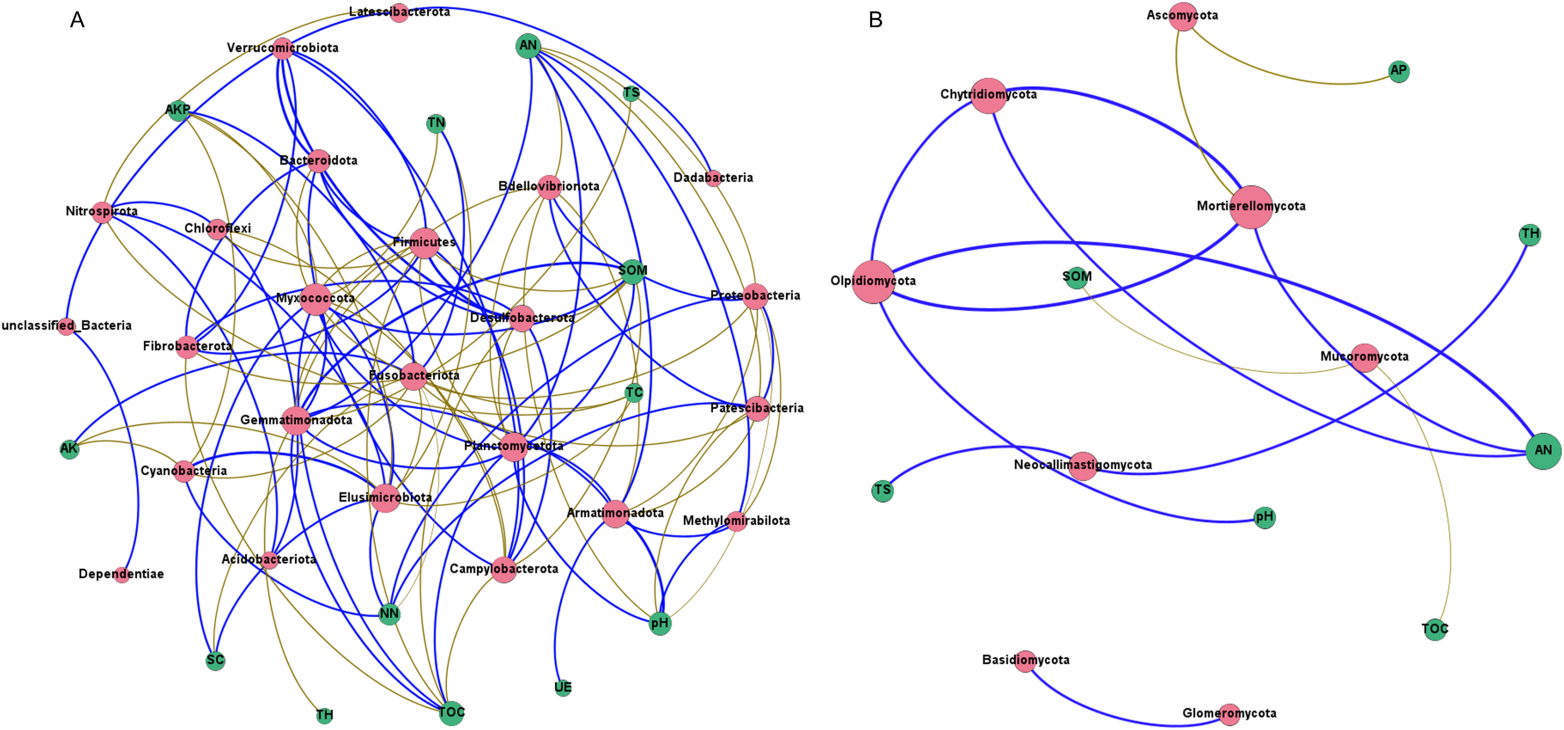
Co-occurrence network of rhizosphere microorganisms (at phylum level) and environmental factors. (A) Bacterial community (magenta circles) and environmental factors (green circles); (B) fungal community (magenta circles) and environmental factors (green circles). Strong correlation (Pearson’s correlation coefficient |R| ≥ 0.6) and significant (*P* < 0.05) results are presented in the network. Blue lines represent positive correlations, gold lines represent negative correlations.

Network analysis was carried out to understand interactions between different rhizosphere microbiota, and between rhizosphere microbiota and environmental factors. It was shown that rhizosphere bacterial community had more complex interaction network than rhizosphere fungal community (Figs. 8A and 8B), which may be attributed to the higher abundance and diversity of the bacterial community. In bacterial community, all 13 environmental factors revealed positive or negative impacts on bacteria at the Phylum level, with more negative (56.60%) than positive correlations (43.40%) were observed (Fig. 8A). Planctomycetota contributed the most to the network, followed by Firmicutes, Myxococcota, and Proteobacteria. In fungal community, only 7 of the 13 environmental factors were associated with rhizosphere fungi (Fig. 8B). AN may be the key factors driving the composition of rhizosphere fungal community. Chytridiomycota, Mortierellomycota, and Olpidiomycota may be the three phyla that had the greatest influences on the fungal community (Fig. 8B). Interestingly, total hydrogen (TH) and total sulfur (TS) were negatively correlated with rhizosphere bacteria but positively correlated with rhizosphere fungi, suggesting that rhizosphere bacteria and fungi respond differently to environmental factors. A co-occurrence network of bacteria-fungi-environmental factors was established (Fig. S2), and more positive interactions (60%) were found in bacteria-bacteria and bacteria-fungi.

## Discussion

A remarkable OTUs variation was found at different development stages in *G. littoralis*. The number of OTUs in both bacteria and fungi communities first decreased and then increased along the seedling-vigorous growth-harvesting development stages. In addition, the proportion of OTUs special to each stage of bacterial and fungal communities also showed similar results, with the values of 14.70–4.81–6.74% and 33.33–20.68–41.46%, respectively. The results may reflect the process of rhizosphere microorganisms colonization. At the seedling stage, due to the weak root system of *G. littoralis*, OTUs may represent indigenous microorganisms in the soil. At the harvesting stage, the rhizosphere microbes adapted to the root exudates are gradually enriched, and community structure become more stable, thus total and special OTUs number increases.

In terms of species abundance, Proteobacteria, Acidobacteria, and Actinobacteria are the main components of bacterial community, and Ascomycota and Basidiomycota are the dominant fungi at each development stage. This is similar to the species composition of the rhizosphere microorganisms in other plants (Nie et al., 2012; Li et al., 2020). The composition of rhizosphere bacteria of *G. littoralis* is also consistent with the endophytic bacterial composition previously reported (Huo et al., 2020). This is as expected, as previous studies have confirmed that most endophytic bacteria come from the soil (Huo et al., 2020). There have been no reports on the composition of endophytic fungi of *G. littoralis*. Proteobacteria play an important role in plant growth and development by promoting photosynthesis to improve the utilization of C-source (Ma et al., 2021). The abundance of Proteobacteria increased significantly from the seedling stage to the vigorous growth stage, but remained stable from the vigorous growth stage to the harvesting stage, which may be related to changes in soil C and N content. Increased abundance of Proteobacteria leads to more efficient use of complex carbohydrates and promotes plant adaptation to high C and N environments. The abundance of Acidobacteria decreased gradually from the seedling stage to the harvesting stage. Acidobacteria mainly degraded plant residues, participated in the metabolism of carbon compounds, and photosynthesis. The reason for the decline of abundance in the late development of *G. littoralis* may be that the microbial community used simple amino acids in the early stage and complex carbohydrates in the late stage. These characteristics have a positive impact on the growth of *G. littoralis*. Ascomycota is the largest fungal group and is important to rhizosphere ecosystems (Beimforde et al., 2014). This group contains a large number of beneficial species (Braun et al., 2012) and harmful species (Peterson et al., 2021). Basidiomycota is an important pathogen lineage in the fungal kingdom (Coelho et al., 2017), and is the main fungi responsible for rust disease of *G. littoralis*. It was relatively abundant at three development stages (9.88–15.2%), with the greatest abundance occurring at the vigorous growth stage, suggesting that the invasion of pathogenic fungi may have occurred at the vigorous growth stage, but *G. littoralis* remained healthy. Previous studies have shown that Proteobacteria and Actinobacteria are important phylum associated with plant disease suppression (Mendes et al., 2011). Therefore, it is speculated that Proteobacteria and Actinobacteria with high abundance may participate in the disease suppression of *G. littoralis* and thus maintain the health of *G. littoralis*. Compared with the previously reported microbial abundance in soils at the harvesting stage with that in continuous crop obstacles soil of *G. littoralis* (Gu et al., 2021), the abundance of Proteobacteria (41.15% *vs* 33%) and Basidiomycota (9.88% *vs* 3.8%) was significantly higher, while that of Actinobacteria (13.66% *vs* 13%) and Acidobacteria (17.94% *vs* 17%) was similar to the former. These results suggest that Proteobacteria with higher abundance may play a key role in disease suppression. In cultivation of *G. littoralis*, controlling the occurrence of diseases and pests is an important prerequisite to ensure the yield. Grasping the occurrence time and abundance of main phytobacteria can provide reference for field management and yield increase at development stages of *G. littoralis*.

The alpha diversity of bacterial community showed a decline with the seedling-vigorous growth-harvesting stage, consistent with results in other species (Shi et al., 2016; Cordovez et al., 2019). The alpha diversity of fungal community decreased first and then increased with the development time. These results indicate that soil type is changing from a bacteria-dominated state to a fungi-dominated stage. However, the Ace, Chao1 and Shannon index of bacteria were significantly higher than those of fungi at all development stages. This suggests that the bacterial species in the rhizosphere soil are more abundant and diverse than fungi. Previous studies have suggested that bacteria-dominated soils are healthier. As the composition of rhizosphere soil changes from a bacteria-dominated state to a fungi-dominated stage, diseases and pests increase, leading to lower crop yield and other adverse effects (Liao et al., 2011). The results presented here indicate that the healthy state of the rhizosphere may be the basis to ensure the high quality of the medicinal herbs of “Beishashen” at the harvesting stage. Combined with the changes in microbial abundance, the difference in alpha diversity index between bacteria and fungi may represent a change trend in soil health status in the late development stage of *G. littoralis*, and the high abundance of Proteobacteria maintained the healthy development of *G. littoralis*.

High functional diversity was observed in the rhizosphere bacterial and fungal communities of *G. littoralis*. For bacterial community, the 10 key functions dominate at all the development stages. Of these, the predictive function of “Membrane transport” was thought to be associated with symbiotic interactions between bacterial community and other organisms (Burke et al., 2011; Ma et al., 2021), suggesting that rhizosphere bacteria may mediate rhizosphere interactions throughout the whole growth process of *G. littoralis*. The functions of “Carbohydrate metabolism”, “Energy metabolism”, and “Amino acid metabolism” are associated with carbon and nitrogen fixation, and rhizosphere bacteria are speculated to convert carbon and nitrogen from soil, thus providing available substances for plants. Through the functional difference analysis, significant differentiations of the functions at the early development stage and the middle to late development stages were observed, which is supported by the results of PCA analysis. The abundance of “environmental adaptation” function at the harvesting stage was significantly higher than that at the seedling stage. This function mainly related to “plant-pathogen interaction”, indicating that the bacterial community may be involved in plant disease resistance at the harvesting stage. For fungal community, functional differentiation also occurred between the early development stage and the middle to late development stages. The relative abundance of saprotroph, symbiotroph, and pathotroph-saprotroph-symbiotroph fungi increased gradually with the growth and development of *G. littoralis*. These fungi may accelerate the decomposition of soil organic matter and convert insoluble minerals in the soil into nutrients available to *G. littoralis*. The results of functional difference analysis also confirmed functional differentiation between early and late development: Only functional changes were found at the seedling stage and vigorous growth stage, and at the vigorous growth stage and harvesting stage, but not at the seedling stage and harvesting stage.

Biomarkers at three development stages were identified using a random forest machine learning algorithm. The 47 bacterial and 22 fungal biomarkers exhibit different correlations at different developmental stages, and these characteristics can be used to distinguish *G. littoralis* at different development stages. Biomarkers may be useful indicators for identifing plant origin, and some studies propose using biomarkers to determine the origin of imported soybeans (Zhou et al., 2022). As a traditional Chinese medicine, the quality of “Beishashen” is of paramount importance. Medicinal herbs grown in a specific area have better efficacy and are called “authentic medicinal herbs”. The samples collected in this study are from the authentic producing areas of *G. littoralis*, therefore this study can provide an option for reliable medicinal herbs origin traceability technology.

The structure and diversity of rhizosphere microorganisms are influenced by multiple environmental factors. The RDA analysis showed that eight environmental factors had significantly influence on the rhizosphere microbial community, and different environmental factors had different influence on microbial community at different development stages. SOM, pH, UE, and AP positively impacted the bacterial and fungal communities at seedling stage, among which the influence of pH on bacterial and fungal communities was the most remarkable, and bacterial community was significantly affected by SOM, while fungal community was significantly impacted by AP. These results demonstrate the importance of pH, SOM and AP in early development of *G. littoralis*. Previous studies have shown that rhizosphere microbial communities promote colonization of beneficial microbes by regulating pH to suppress plant immunity (Yu et al., 2019). This suggests that pH may play an important role in rhizosphere microorganisms colonization at the seedling stage of *G. littoralis*. SOM and AP are important indicators of soil fertility, and their effects on early plant rhizosphere need further study. SC and NN showed significant positive effects on bacterial and fungal communities at the vigorous growth stage and harvesting stage. SC can hydrolyse sucrose in soil to produce small molecules of glucose, which is an important carbon source for most microbes. NN is an important nitrogen source for most microorganisms. Therefore, it can be inferred that rhizosphere microbes absorbs a lot of carbon and nitrogen sources and carried out vigorous metabolic activities at the vigorous growth stage and harvesting stage of *G. littoralis*. The metabolites produced can provide available nutrients for *G. littoralis* and promote the production of active secondary metabolites. RDA analysis also provides a list of the bacteria and fungi that play a positive role in different development stages, so the microorganisms that are beneficial to the growth of *G. littoralis* can be screened. Isolation and culture of these beneficial microorganisms are of great significance to improve the yield and quality of *G. littoralis*. Understanding the influence of various environmental factors on rhizosphere microbial community can help us to construct beneficial microbiome by regulating environmental factors to promote the healthy growth of plants and improve the yield of medicinal herbs.

Co-occurrence networks can identify putative interactions between microorganisms in the environment (Berry & Widder, 2014). The results revealed more complex co-occurrence networks in bacterial community than in fungal community. The formation of bacterial community involves more environmental factors than the formation of fungal community. Other studies have found complex co-occurrence networks in rhizosphere bacterial communities (Fan et al., 2017; Zhang et al., 2022). Some studies have shown that rhizosphere influences ultimately lead to a decrease in bacterial community diversity and more complex symbiotic networks (Shi et al., 2016; Cordovez et al., 2019). Our findings support this conclusion. In order to understand all rhizosphere microbial interactions, a co-occurrence network of bacteria-fungi-environmental factors was established. More positive interactions (60%) in bacteria-bacteria and bacteria-fungi were found. The results suggest that the rhizosphere microorganisms form a complex mutualistic symbiosis network, which might be beneficial to the growth and development of *G. littoralis*. These results are also consistent with previous studies showing that most bacteria cluster together as functional groups, which use plant-derived resources more efficiently and provide a greater number of services (Saleem, Hu & Jousset, 2019).

## Conclusions

In this study, high-throughput sequencing technology revealed the dynamic changes of rhizosphere microbial communities (bacteria and fungi) at different development stages of *G. littoralis*. The composition, diversity and function of rhizosphere bacterial and fungal communities are closely related to the development stage of *G. littoralis*. Eight environmental factors play a vital role in driving rhizosphere microbial changes at different development stages. This study provides data support for understanding the structure and composition of the rhizosphere microbial community in the development period of *G. littoralis*, and also lays a foundation for improving the yield and quality of *G. littoralis* by regulating the microbial community in the future. There are still many questions to be explored about how to utilize rhizosphere microbial resources, how to increase microbial diversity in agro-ecosystems, and the mechanisms of microbial diversity contribute to agricultural production.

## Supplemental Information

10.7717/peerj.14988/supp-1 Supplemental Information 1 Principal component analysis (PCA) based on the functional abundance of rhizosphere microorganisms. (A) Bacterial function at the level of class 2 kegg pathway; (B) bacterial function at the level of class 3 kegg pathway; (C) fungal distribution based on trophic modes;

10.7717/peerj.14988/supp-2 Supplemental Information 2 Co-occurrence network of rhizosphere of Bacteria, Fungi and environmental factors. Purple circles represent Bacteria; Green circles represent Fungi; Magenta circles represent environmental factors.

10.7717/peerj.14988/supp-3 Supplemental Information 3 Relative abundance of bacteria at phylum level at each development stage.

10.7717/peerj.14988/supp-4 Supplemental Information 4 Relative abundance of fungi at phylum level at each development stage.

10.7717/peerj.14988/supp-5 Supplemental Information 5 Percentage of top ten functions of rhizosphere bacteria in *G. litteralis*. Ss, the seedling stage; Vs, the vigorous growth stage; Hs, the harvesting stage.

10.7717/peerj.14988/supp-6 Supplemental Information 6 Percentage of rhizosphere fungi of different trophic modes in *G. litteralis*. Ss, the seedling stage; Vs, the vigorous growth stage; Hs, the harvesting stage.

10.7717/peerj.14988/supp-7 Supplemental Information 7 Raw data for soil physiochemical properties (environmental factors). Ss, the seedling stage; Vs, the vigorous growth stage; Hs, the harvesting stage. AP, available phosphorus; AK, available potassium; AN, ammonium nitrogen; SOM, soil organic matter; TOC, total organic carbon; NN, nitrate nitrogen; SC, Saccharase; UE, Urease; AKP, alkaline phosphatase; TN, total nitrogen; TH, total hydrogen; TC, total carbon; TS, total sulfur.

## Additional Information and Declarations

### Field Study Permissions

The following information was supplied relating to field study approvals (*i.e*., approving body and any reference numbers): Field experiments were approved by the Ludong University (project number 20210301).

### Data Availability

The following information was supplied regarding data availability: The sequences are available at GenBank: PRJNA903756. The raw data is available in the Supplemental Files.

## References

[ref-1] Badri DV, Zolla G, Bakker MG, Manter DK, Vivanco JM (2013). Potential impact of soil microbiomes on the leaf metabolome and on herbivore feeding behavior. New Phytologist.

[ref-2] Bai B, Liu W, Qiu X, Zhang J, Zhang J, Bai Y (2022). The root microbiome: community assembly and its contributions to plant fitness. Journal of Integrative Plant Biology.

[ref-3] Bao SD (2000). Soil and agricultural chemistry analysis.

[ref-4] Bardou P, Mariette J, Escudié F, Djemiel C, Klopp C (2014). jvenn: an interactive Venn diagram viewer. BMC Bioinformatics.

[ref-5] Bastian M, Heymann S, Jacomy M (2009). Gephi: an open source software for exploring and manipulating networks. Third International AAAI Conference on Weblogs and Social Media.

[ref-6] Beimforde C, Feldberg K, Nylinder S, Rikkinen J, Tuovila H, Dörfelt H, Gube M, Jackson DJ, Reitner J, Seyfullah LJ, Schmidt AR (2014). Estimating the Phanerozoic history of the Ascomycota lineages: combining fossil and molecular data. Molecular Phylogenetics and Evolution.

[ref-7] Berry D, Widder S (2014). Deciphering microbial interactions and detecting keystone species with co-occurrence networks. Frontiers in Microbiology.

[ref-8] Bolger AM, Lohse M, Usadel B (2014). Trimmomatic: a flexible trimmer for Illumina sequence data. Bioinformatics.

[ref-9] Bolyen E, Rideout JR, Dillon MR, Arumugam M, Asnicar F, Bai Y, Bisanz JE, Bittinger K, Brejnrod A, Colin J, Brislawn C, Brown T, Benjamin J, Callahan A (2019). Reproducible, interactive, scalable and extensible microbiome data science using QIIME 2. Nature Biotechnology.

[ref-10] Braun SE, Sanderson JP, Daughtrey ML, Wraight SP (2012). Attraction and oviposition responses of the fungus gnat *Bradysia* impatiens to microbes and microbe-inoculated seedlings in laboratory bioassays. Entomologia Experimentalis et Applicata.

[ref-11] Breiman L, Last M, Rice J (2006). Random forests: finding quasars. Statistical Challenges in Astronomy.

[ref-12] Burke C, Steinberg P, Rusch D, Kjelleberg S, Thomas T (2011). Bacterial community assembly based on functional genes rather than species. Proceedings of the National Academy of Sciences of the United States of America.

[ref-13] Caporaso JG, Lauber CL, Walters WA, Berg-Lyons D, Lozupone CA, Turnbaugh PJ, Fierer N, Knight R (2011). Global patterns of 16S rRNA diversity at a depth of millions of sequences per sample. Proceedings of the National Academy of Sciences of the United States of America.

[ref-14] Cesarano G, Zotti M, Antignani V, Marra R, Scala F, Bonanomi G (2017). Soil sickness and negative plant-soil feedback: a reappraisal of hypotheses. Journal of Plant Pathology.

[ref-15] Chen T, Liu Y, Huang L (2022). ImageGP: an easy-to-use data visualization web server for scientific researchers. iMeta.

[ref-16] Coelho MA, Bakkeren G, Sun S, Hood ME, Giraud T (2017). Fungal sex: the *Basidiomycota*. The Fungal Kingdom.

[ref-17] Cordovez V, Dini-Andreote F, Carrión VJ, Raaijmakers JM (2019). Ecology and evolution of plant microbiomes. Annual Review of Microbiology.

[ref-18] Douglas GM, Maffei VJ, Zaneveld J, Yurgel SN, Brown JR, Taylor CM, Huttenhower C, Langille MGI (2019). PICRUSt2: an improved and extensible approach for metagenome inference. BioRxiv.

[ref-19] Edgar RC (2013). UPARSE: highly accurate OTU sequences from microbial amplicon reads. Nature Methods.

[ref-20] Edgar RC, Haas BJ, Clemente JC, Quince C, Knight R (2011). UCHIME improves sensitivity and speed of chimera detection. Bioinformatics.

[ref-21] Fan K, Cardona C, Li Y, Shi Y, Xiang X, Shen C, Wang H, Gilbert JA, Chu H (2017). Rhizosphere-associated bacterial network structure and spatial distribution differ significantly from bulk soil in wheat crop fields. Soil Biology and Biochemistry.

[ref-22] Fu L (1992). Red data book of plants in China.

[ref-23] Gardes M, Bruns TD (1993). ITS primers with enhanced specificity for basidiomycetes - application to the identification of mycorrhizae and rusts. Molecular Ecology.

[ref-24] Gouda S, Kerry RG, Das G, Paramithiotis S, Shin HS, Patra JK (2018). Revitalization of plant growth promoting rhizobacteria for sustainable development in agriculture. Microbiological Research.

[ref-25] Gu J, Li Y, Sun H, Zheng Q, Yan Y, Han X (2021). Effects of continuous cropping on soil nutrients,enzyme activities and microbial community diversity of *Glehnia littoralis*. Journal of Chinese Medicinal Materials.

[ref-26] Guan S (1986). Soil enzyme and its method.

[ref-27] Huo X, Wang Y, Zhang D, Gao T, Liu M (2020). Characteristics and diversity of endophytic bacteria in endangered Chinese herb *Glehnia littoralis* based on illumina sequencing. Polish Journal of Microbiology.

[ref-28] Huse SM, Dethlefsen L, Huber JA, Welch DM, Relman DA, Sogin ML (2008). Exploring microbial diversity and taxonomy using SSU rRNA hypervariable tag sequencing. PLOS Genetics.

[ref-29] Ihaka R, Gentleman R (1996). R: a language for data analysis and graphics. Journal of Computational and Graphical Statistics.

[ref-30] Jing Y, Li J, Zhang Y, Zhang R, Zheng Y, Hu B, Wu L, Zhang D (2021). Structural characterization and biological activities of a novel polysaccharide from *Glehnia littoralis* and its application in preparation of nano-silver. International Journal of Biological Macromolecules.

[ref-31] Karthikeyan B, Jaleel CA, Lakshmanan GMA, Deiveekasundaram M (2008). Studies on rhizosphere microbial diversity of some commercially important medicinal plants. Colloids and Surfaces B: Biointerfaces.

[ref-32] Khamna S, Yokota A, Lumyong S (2009). Actinomycetes isolated from medicinal plant rhizosphere soils: diversity and screening of antifungal compounds, indole-3-acetic acid and siderophore production. World Journal of Microbiology and Biotechnology.

[ref-35] Köberl M, Schmidt R, Ramadan EM, Bauer R, Berg G (2013). The microbiome of medicinal plants: diversity and importance for plant growth, quality, and health. Frontiers in Microbiology.

[ref-33] Kremer RJ (2006). Deleterious rhizobacteria. Plant-Associated Bacteria.

[ref-34] Kushwaha RK, Rodrigues V, Kumar V, Patel H, Raina M, Kumar D, Varma A, Tripathi S, Prasad R (2020). Soil microbes-medicinal plants interactions: ecological diversity and future prospect. Plant Microbe Symbiosis.

[ref-36] Li J, Luo Z, Zhang C, Qu X, Chen M, Song T, Yuan J (2020). Seasonal variation in the rhizosphere and non-rhizosphere microbial community structures and functions of *Camellia yuhsienensis* Hu. Microorganisms.

[ref-37] Liao HB, Li YX, Shao JJ, Fang F, Guo WD, Chen WR (2011). Impacts of continuous cropping on *Fritillaria thunbergii* Miq. growth and rhizosphere soil properties. Chinese Journal of Ecology.

[ref-38] Lu T, Ke M, Lavoie M, Jin Y, Fan X, Zhang Z, Fu Z, Sun L, Gillings M, Peñuelas J, Qian H, Zhu YG (2018). Rhizosphere microorganisms can influence the timing of plant flowering. Microbiome.

[ref-39] Ma Z, Yi Z, Bayar K, Fu Y, Liu H (2021). Community dynamics in rhizosphere microorganisms at different development stages of wheat growing in confined isolation environments. Applied Microbiology and Biotechnology.

[ref-40] Magoc T, Salzberg SL (2011). FLASH: fast length adjustment of short reads to improve genome assemblies. Bioinformatics.

[ref-41] Martin M (2011). Cutadapt removes adapter sequences from high-throughput sequencing reads. EMBnet.journal.

[ref-42] Mendes R, Kruijt M, de Bruijn I, Dekkers E, van der Voort M, Schneider JHM, Piceno YM, DeSantis TZ, Andersen GL, Bakker PAHM, Raaijmakers JM (2011). Deciphering the rhizosphere microbiome for disease-suppressive bacteria. Science.

[ref-43] Nguyen NH, Song Z, Bates ST, Branco S, Tedersoo L, Menke J, Schilling JS, Kennedy PG (2016). FUNGuild: an open annotation tool for parsing fungal community datasets by ecological guild. Fungal Ecology.

[ref-44] Nie M, Meng H, Li K, Wan JR, Quan ZX, Fang CM, Chen JK, Li B (2012). Comparison of bacterial and fungal communities between natural and planted pine forests in subtropical China. Current Microbiology.

[ref-45] Parks DH, Tyson GW, Hugenholtz P, Beiko RG (2014). STAMP: statistical analysis of taxonomic and functional profiles. Bioinformatics.

[ref-46] Peterson D, Li T, Calvo AM, Yin Y (2021). Categorization of orthologous gene clusters in 92 *Ascomycota* genomes reveals functions important for phytopathogenicity. Journal of Fungi.

[ref-47] Quast C, Pruesse E, Yilmaz P, Gerken J, Schweer T, Yarza P, Peplies J, Glöckner FO (2013). The SILVA ribosomal RNA gene database project: improved data processing and web-based tools. Nucleic Acids Research.

[ref-48] Racine JS (2012). RSTUDIO: a platform-independent IDE for R and sweave. Journal of Applied Econometrics.

[ref-49] Razavi BS, Zarebanadkouki M, Blagodatskaya E, Kuzyakov Y (2016). Rhizosphere shape of lentil and maize: spatial distribution of enzyme activities. Soil Biology and Biochemistry.

[ref-50] Saleem M, Hu J, Jousset A (2019). More than the sum of its parts: microbiome biodiversity as a driver of plant growth and soil health. Annual Review of Ecology, Evolution, and Systematics.

[ref-51] Shan R, She M (1992). Flora of China.

[ref-52] Shi S, Nuccio EE, Shi ZJ, He Z, Zhou J, Firestone MK (2016). The interconnected rhizosphere: high network complexity dominates rhizosphere assemblages. Ecology Letters.

[ref-53] Solaiman ZM, Anawar HM (2015). Rhizosphere microbes interactions in medicinal plants. Plant-Growth-Promoting Rhizobacteria (PGPR) and Medicinal Plants.

[ref-54] Sun B, Song G, Zheng N, Zhang Y, Chen A, Yun F, Yong H, Zong H, Qiao Y, Zhang Y, He N, Zheng Z (2016). Determination of nitrate nitrogen in soil. Ultraviolet spectrophotometry method.

[ref-55] Taesook Y, Myeong SC, Lee AY, Do YL, Byeong CM, Jin MC, Byung KC, Ho KK (2010). Anti-inflammatory activity of methylene chloride fraction from *Glehnia littoralis* extract via suppression of NF-κB and mitogen-activated protein kinase activity. Journal of Pharmacological Sciences.

[ref-56] Trivedi P, Leach JE, Tringe SG, Sa T, Singh BK (2020). Plant-microbiome interactions: from community assembly to plant health. Nature Reviews Microbiology.

[ref-57] Vacheron J, Desbrosses G, Bouffaud M-L, Touraine B, Moënne-Loccoz Y, Muller D, Legendre L, Wisniewski-Dyé F, Prigent-Combaret C (2013). Plant growth-promoting rhizobacteria and root system functioning. Frontiers in Plant Science.

[ref-58] White TJ, Bruns T, Lee S, Taylor J (1990). Amplification and direct sequencing of fungal ribosomal RNA genes for phylogenetics. PCR Protocols.

[ref-59] Xiong C, Singh BK, He JZ, Han YL, Li PP, Wan LH, Meng GZ, Liu SY, Wang JT, Wu CF, Ge AH, Zhang LM (2021). Plant developmental stage drives the differentiation in ecological role of the maize microbiome. Microbiome.

[ref-60] Yu K, Liu Y, Tichelaar R, Savant N, Lagendijk E, van Kuijk SJL, Stringlis IA, van Dijken AJH, Pieterse CMJ, Bakker PAHM, Haney CH, Berendsen RL (2019). Rhizosphere-associated *Pseudomonas* suppress local root immune responses by gluconic acid-mediated lowering of environmental pH. Current Biology.

[ref-61] Yuan Y, Zuo J, Zhang H, Zu M, Liu S (2022). The Chinese medicinal plants rhizosphere: metabolites, microorganisms, and interaction. Rhizosphere.

[ref-62] Zakry FAA, Shamsuddin ZH, Khairuddin AR, Zin ZZ, Anuar AR (2012). Inoculation of *Bacillus sphaericus* UPMB-10 to young oil palm and measurement of its uptake of fixed nitrogen using the 15n isotope dilution technique. Microbes and Environments.

[ref-63] Zhang Z, Chai X, Gao Y, Zhang B, Lu Y, Huang C, Li L, Tariq A, Li X, Zeng F (2022). Dynamics in diversity, co-occurrence pattern, and community assembly of a perennial desert plant root-associated bacteria. Rhizosphere.

[ref-64] Zhou X, Zhang H-L, Lu X-W, Zhao P, Liu F, Qi Z-H, Tang F, Duan W-J, Cai L (2022). Applying meta-data of soybean grain in origin trace and quarantine inspection. Food Research International.

