# Supplementary figures and images for: Community differentiation of rhizosphere microorganisms and their responses to environmental factors at different development stages of medicinal plant Glehnia littoralis
Source: PeerJ. 2023 Mar 6;11:e14988. doi: 10.7717/peerj.14988 (PMC9997192; doi:10.7717/peerj.14988)

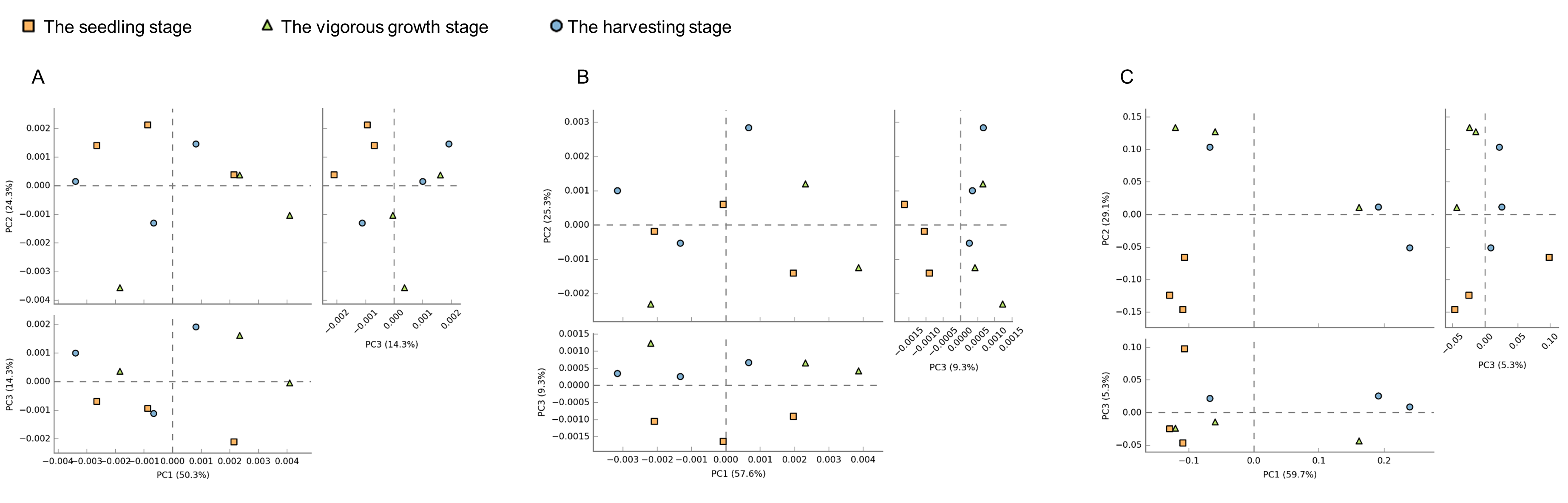

Supplement: Supplemental Information 1 — (A) Bacterial function at the level of class 2 kegg pathway; (B) bacterial function at the level of class 3 kegg pathway; (C) fungal distribution based on trophic modes; [file peerj-11-14988-s001.png]

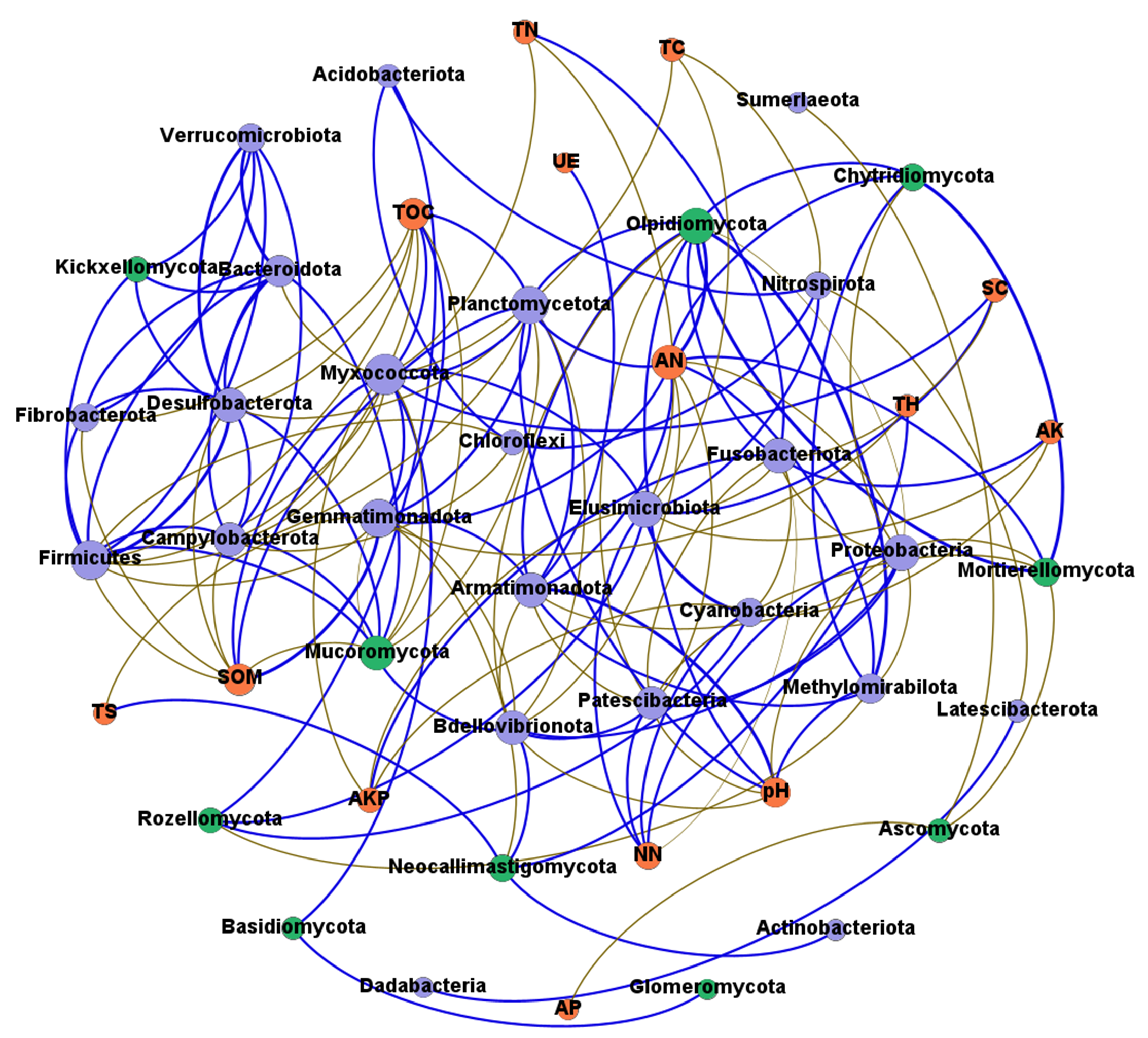

Supplement: Supplemental Information 2 — Purple circles represent Bacteria; Green circles represent Fungi; Magenta circles represent environmental factors. [file peerj-11-14988-s002.png]
